# High School Exiting Among Autistic Students: A National Analysis of Special Education Data from 2015 to 2019

**DOI:** 10.3390/bs16040566

**Published:** 2026-04-09

**Authors:** Kiley J. McLean, Meghan E. Carey, Dylan Cooper, Kristen Lyall, David S. Mandell, Lindsay L. Shea

**Affiliations:** 1 Helen Bader School of Social Welfare, University of Wisconsin-Milwaukee, Milwaukee, WI 53211, USA; 2 Penn Center for Mental Health, Perelman School of Medicine, University of Pennsylvania, Philadelphia, PA 19104, USA; 3 A.J. Drexel Autism Institute, Drexel University, Philadelphia, PA 19104, USA

**Keywords:** autism, intellectual disability, school-based interventions, transition planning, special education, transition and employment

## Abstract

The Individuals with Disabilities Education Act (IDEA) provides special education services to students with disabilities, including autistic students, until age 21. However, the ages at which autistic students exit high school—and the reasons for exit—are not well documented, despite their importance for transition planning. We analyzed U.S. Department of Education Section 618 Part B data for special education students ages 14–21 across five school years (2014–2015 to 2018–2019) to examine exit age and exit category, with comparisons among autistic students, students with intellectual disabilities (IDs), and students with other disabilities. Using publicly reported counts of students exiting at each age, we derived mean exit ages by transforming age-specific count data. In 2019, 71% of autistic students graduated with a diploma, compared with 48% of students with IDs and 72.5% of students with other disabilities. Autistic students had lower dropout rates (6–8%) than students with other disabilities (15–18%). The mean exit age for autistic students was approximately 18 years, with an average graduation age of 17.9 years, indicating that many students exited prior to the end of extended IDEA eligibility in their state. These findings provide descriptive context on when autistic students exit high school relative to IDEA eligibility and underscore the importance of transition planning and coordination with adult service systems, though these factors were not directly examined in the present analysis.

## 1. Introduction

The Individuals with Disabilities Education Act (IDEA) ensures special education services up to age 21 for students with disabilities, including autistic students (U.S. Department of Education [DOE], 2023b; Lipkin et al., 2015). IDEA includes early intervention for young children with developmental delays, transition services from high school to adulthood, and preparation for employment and community involvement (Katsiyannis et al., 2001). While this federal entitlement aims to support equitable educational access and outcomes, significant differences exist in how students with various disabilities utilize these services and their outcomes beyond high school (Hughes et al., 2023; Anderson et al., 2019; Katsiyannis et al., 2001). Understanding differences in IDEA utilization is crucial for addressing disparities in transition outcomes, particularly given that students with autism, intellectual disability, and other eligibility categories often exhibit unique challenges and strengths throughout the course of their lives (Anderson et al., 2019; Taylor & Seltzer, 2011; Wehman et al., 2014).

Since 1990, a significant focus has been on transition services, defined as a coordinated set of activities aimed at enhancing academic and functional achievements to facilitate the move from school to various post-school activities (Hughes et al., 2023; U.S. Department of Education [DOE], 2023b). Under IDEA, students with disabilities are entitled to special education services past the typical graduation age of 18, up to age 21, which assists in further preparation for life after school (LD Online, 2023). At the same time, the appropriateness and use of extended eligibility can vary depending on students’ individualized transition goals, educational pathways, and local district practices. However, a key stipulation of U.S. federal law (20 U.S.C. § 1412(a)(1)(B)(i)) asserts that this obligation does not apply if it conflicts with state law or practice. A recent 9th Circuit ruling (N.D. v. Reykdal, No. 23-35580, 9th Cir. 2024) clarified that states have the discretion to cap services at age 18, provided this is uniformly applied to all students, irrespective of disability. This has led to diverse state-level approaches to extending special education services, which may contribute to variation in when students exit high school and whether they remain enrolled to access extended eligibility. Understanding these exit patterns is therefore important for interpreting how policy context may shape transition experiences across states (LD Online, 2023).

The need for high-quality transition planning is also underscored by the well-documented phenomenon of autistic students “dropping off the service cliff” after high school, where they often become disengaged from community activities and services such as education and employment (Kirby et al., 2016; Laxman et al., 2019; Roux et al., 2015). This phenomenon is exacerbated by systemic gaps in services for adults and inconsistency in adult support systems (Laxman et al., 2019). Evidence suggests that autistic students may be less likely to drop out of high school compared to their peers with other disabilities, but they also face these unique challenges during the transition to adulthood, leading to lower rates of post-secondary education and employment (Taylor & Seltzer, 2011; Roux et al., 2020). While the present study does not directly measure post-school outcomes, examining patterns in exit age and exit category provides important descriptive context for understanding transition timing within this broader landscape. Importantly, exiting high school at earlier or later ages does not necessarily represent a positive or negative outcome; rather, these patterns may reflect a range of individualized educational pathways, support needs, and district-level practices. Differences in exit rates and graduation ages across disability categories may reflect differences in educational pathways and support needs; they may also relate to variation in access to transition planning and support systems, which warrants further investigation in future research.

This study contributes to the literature by providing a detailed analysis of high school exit patterns and graduation ages among autistic students, those with intellectual disabilities, and other disability groups, offering a national descriptive perspective on how students exit special education systems under IDEA. Students with intellectual disabilities were examined as a comparison group because they often experience extended educational timelines and transition planning structures that differ from those of autistic students, while the broader “other disabilities” category provides a descriptive benchmark for the overall population of students receiving special education services. Although this “other disabilities” category is heterogeneous, it is included to contextualize patterns among autistic students rather than to serve as a homogeneous analytic comparison group. Accordingly, comparisons involving this group should be interpreted cautiously and not as reflecting differences attributable to specific disability subgroups.

Specifically, this study addresses the following research questions: (1) What are the patterns of high school exit categories among autistic students compared with students with intellectual disabilities and students with other disabilities? and (2) How does the average age of graduating with a regular diploma among autistic students vary across U.S. states? By characterizing these national patterns, this study provides a foundation for future research and policy efforts aimed at improving transition supports and outcomes for autistic students. This inquiry is crucial in a landscape where the expanding definition of autism demands more nuanced and adaptable support systems to accommodate the diverse needs of autistic students.

## 2. Materials and Methods

We conducted a repeated cross-sectional analysis for school years 2014–2019 (five school years) using publicly available data from the US Department of Education (https://data.ed.gov/dataset, accessed 1 September 2025). Section 618 of the Individuals with Disabilities Education Act (IDEA) requires each US state and territory to submit data about the students with disabilities who receive special education services to the US Department of Education (Part B; U.S. Department of Education [DOE], 2023a, accessed 1 September 2025). These data include individuals aged 6–21 receiving services at public expense across educational settings, including public schools, correctional facilities, and state-operated programs. Data from “Exiting” files include information about special education students ages 14–21 and provide the reason for their exit from special education (U.S. Department of Education [DOE], 2022). These data are de-identified and freely accessible, and IRB approval was not required for this study. Because the dataset consists of aggregated administrative counts rather than a sampled population, analyses were descriptive and not weighted or adjusted for state population size.

Outcomes of interest were the reason for exiting high school, as described in the IDEA data (hereafter “Exit Category”), and the mean age of exiting. There are five exit categories of interest: (1) died; (2) dropped out; (3) graduated with a regular diploma; (4) graduated with a certificate; and (5) reached a maximum age but did not receive a diploma (the maximum age to which special education services are provided varies by state). According to IDEA Part B Exiting data for the 2018–2019 school year, most states’ maximum age was either 21 or 22 (n = 43), with four states reporting a maximum age of 20, and two states reporting a maximum age of 26. We excluded two exit categories indicating continued high school enrollment: (1) “moved, known to be continuing,” and (2) “transferred to regular education.” These outcome categories were outside the scope of the current study, which focused on students leaving high school. In the 2017–2018 school year, IDEA introduced an additional exiting category called “Graduated with alternate high school diploma.” No states reported data for this category in the 2017–2018 or 2018–2019 school years, and it was excluded from analyses.

The age variable in the IDEA exiting files reflects students’ chronological age at the time of exit from special education services, with counts reported for each age category between 14 and 21 years. To estimate mean exit age, we calculated a weighted mean by multiplying the number of students exiting at each age by that age, summing across age categories, and dividing by the total number of students in the exit category. Cells with suppressed or missing counts were excluded from calculations. Because suppression is more likely in states with smaller populations or specific exit categories, this approach may introduce estimation bias if missingness is not random. Accordingly, mean age estimates should be interpreted cautiously, particularly for states with incomplete data.

For the first aim of this study, we described the exiting patterns among autistic students and students with intellectual disability and compared that to students with any other disability (e.g., exclusive of autism and intellectual disability). The separation of autistic students from those with intellectual disabilities allows for a nuanced understanding of the unique transitional needs and challenges faced by each group, providing insights that could lead to more targeted and effective interventions. Students categorized as “other disabilities” include all remaining IDEA eligibility categories (e.g., deaf-blindness, hearing impairment, specific learning disability, and others), which were aggregated to provide a comparison group representing the broader population of students receiving special education services. While this grouping includes heterogeneous disability categories, it was used to provide a descriptive benchmark rather than to make inferences about specific disability subgroups.

We calculated the proportion exiting special education for each of the exit categories provided in the IDEA data by graduating year. We present the proportions separately by disability category (autism, intellectual disability) and for all students with disabilities (all disabilities). For the second aim of this study, we compared the mean age of graduating with a regular diploma across each of the 50 U.S. states and the District of Columbia. Like other studies analyzing special education data, we excluded territories from our analysis due to the unique historical and contemporary contexts within territories that we were unable to capture in our data (Kurth et al., 2014). As a result, the findings primarily reflect patterns within U.S. states and the District of Columbia and may not fully generalize to U.S. territories. Sensitivity analyses, including territories, could not be conducted at this time.

The IDEA exiting files do not include demographic characteristics (e.g., race, ethnicity, and socioeconomic indicators) at the disability category level; therefore, these variables were not included. Additionally, variation in state reporting practices across states and years may affect comparability, including differences in classification, reporting thresholds, or data suppression. Certificate programs may also differ across states and districts, limiting the interpretation of these outcomes. Because the dataset consists of aggregated administrative counts without individual-level covariates, clustering, or variance estimates, more advanced statistical approaches (e.g., regression modeling or trend testing) were not appropriate. Accordingly, analyses were intentionally descriptive, consistent with the limited prior reports using IDEA Section 618 data, and do not support statistical inference (IDEA Data Center [IDC], n.d., https://ideadata.org/618Reporting/, accessed 1 September 2025).

## 3. Results

**Figure 1:**
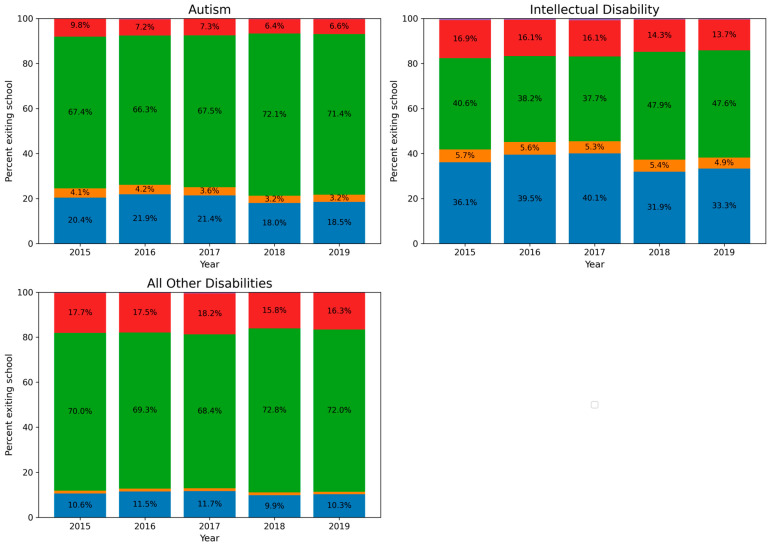
Annual proportion of special education students exiting school by IDEA exit category, 2016–2019. Percentages are displayed within stacked bars; labels for small segments may overlap. Percentages may not sum to 100% due to rounding. Note. Figures distinguish autism, intellectual disability, and all other IDEA-specified disabilities (includes deaf-blindness, emotional disturbance, hearing impairment, multiple disabilities, orthopedic impairment, other health impairment, specific learning disability, speech or language impairment, traumatic brain injury, and visual impairment; excludes autism and intellectual disability). Exit categories include graduated with a regular diploma, dropped out, received a certificate, reached maximum age, and died. Percentages within bars indicate the proportion of students exiting in each category.

The proportion of special education students exiting school by each exit category is presented in Figure 1. Autistic students made up roughly 7%, and those with an intellectual disability made up roughly 10% of all special education students exiting high school across the study period. In all disability categories (autism, intellectual disability, and all other disabilities), the number of students exiting increased during the study period. Because the IDEA Section 618 data are administrative counts and reporting practices may vary across years and states, the reasons for this increase cannot be determined from the available data, but may reflect changes in reporting practices, cohort size, or other policy or demographic factors at the state level. Changes in autism identification practices or eligibility patterns during the study period may also contribute to increases in the number of autistic students represented in IDEA administrative reporting. The increase in autistic students exiting was greater than for those with intellectual disability.

The proportion of autistic students graduating with a regular high school diploma also increased over the study period (2015: 67.4%, 2019: 71.4%). This proportion was higher than that of students with intellectual disability consistently across the study period (e.g., 2019: 47.6%) but was lower than that of students with other disabilities (2019: 74.5%). A higher proportion of autistic students and students with intellectual disability received a certificate when exiting school compared to students with other disabilities (2019: 18.5%, 33.3%, and 7.2%, respectively). Certificates and diplomas represent different types of educational completion within special education systems and may correspond to different postsecondary pathways, although these outcomes cannot be directly examined using the available dataset. Dropout rates for autistic students ranged from 6 to 8% during the study period.

Throughout the study period, autistic students were less likely to drop out compared to students with intellectual disability or students with other disabilities (intellectual disability: 14–17%, other disabilities: 17–20%). These comparisons are descriptive, and no statistical significance testing or variance estimates were calculated. These comparisons do not account for differences in state population size or cohort composition and should therefore be interpreted as descriptive rather than directly comparable across states. The analyses were designed to summarize patterns in administrative data using descriptive statistics (e.g., proportions and mean ages), which are appropriate for presenting overall trends and distributions in large datasets rather than testing statistical inference. A higher proportion of students with intellectual disability (4.9%) and autism (3.2%) reached the maximum age for special education compared to students with other disabilities (0.5%).

Because small cell counts are suppressed in IDEA reporting to protect student privacy, NA values appear for some states in Table 1. These suppressed values may introduce bias if missingness is systematically associated with state size or reporting practices.

Table 1 displays the average age when exiting special education programming among those graduating with a diploma by state. States with an average graduation age of 19 years or older are highlighted to illustrate locations where, on average, students remained enrolled beyond the typical high school graduation age of 18. This threshold is used descriptively to identify states where average exit timing extends into an additional year of IDEA eligibility and does not reflect a formal analytic cutoff. Only three U.S. states had an average age of graduating with a diploma of 19 years or older (New Jersey, Washington, and Wyoming). Autistic students receiving special education services in all other states graduated with a diploma between the ages of 17 and 18. The average age for students with intellectual disabilities graduating with a diploma was higher than that of autistic students in 41 of the 43 states that provided data on age at graduation. These state-level comparisons are presented descriptively, and no formal statistical comparisons across states were conducted.

**Figure 2:**
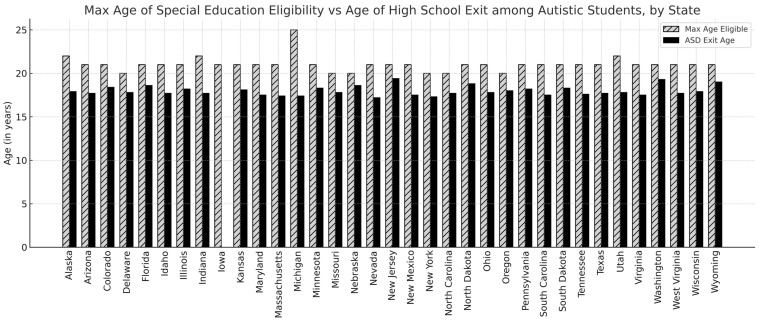
Maximum age of special education eligibility versus average age of high school exit among autistic students, by state.

Figure 2 graphically compares the maximum age of eligibility to the average age of high school exit among autistic adults for each state. This visual comparison provides descriptive context for how variation in state eligibility policies aligns with observed exit ages; however, no formal correlation or regression analysis was conducted. Because state-level estimates represent aggregated administrative reporting, these comparisons should be interpreted descriptively and do not account for clustering, weighting, population size, or other policy differences across states, and therefore, should not be interpreted as causal or directly comparable across states.

## 4. Discussion

Using nationally reported IDEA Section 618 administrative data, this study provides a descriptive overview of high school exit patterns among autistic students across the United States. The findings highlight differences in exit age and exit categories across disability groups and states, offering insight into how students transition out of special education systems. This study provides information about how autistic students utilize their IDEA educational entitlements, revealing that only a small fraction (3.2–4.2%) remain enrolled in special education until reaching the maximum age of IDEA eligibility in the dataset. The average graduation age for autistic students was 17.9 years in 2019, even in states with higher age limits, suggesting that many students exit prior to the upper age limits permitted under IDEA. Decisions about when students exit special education likely reflect a range of individualized factors, including student preferences, family priorities, and Individualized Education Program (IEP) team processes; however, these factors are not captured in the aggregated administrative data used in this study. Extended eligibility may support some students’ transition goals, while others may pursue earlier graduation to access postsecondary opportunities or employment.

In Michigan, where the age limit is 25, most students exited before turning 18. These descriptive patterns suggest that policy environments and graduation structures may influence exit timing across states, although these mechanisms cannot be directly tested using the available data. From 2015 to 2019, the proportion of autistic students graduating with a regular diploma increased by 4%, while those with intellectual disabilities were less likely to graduate with a diploma and more likely to remain in school until the maximum age. This descriptive increase over the study period suggests a modest upward trend in diploma attainment among autistic students in IDEA administrative reporting, although the aggregated dataset does not allow for formal modeling of longitudinal changes. Additionally, autistic students (18.5%) and those with intellectual disabilities (33.3%) were more likely than peers with other disabilities (7.2%) to exit high school with certificates. Because certificate programs vary widely across states and districts, these outcomes may reflect differences in educational pathways, access to diploma tracks, or district-level graduation policies.

The higher percentage of autistic students graduating with a diploma and their lower dropout rate compared to other disabilities may reflect differences in educational pathways, support needs, or eligibility characteristics across disability categories. Because the IDEA administrative dataset used in this study does not include indicators of transition planning quality or school-level programming, the present analysis cannot determine the specific factors contributing to these differences. Other contextual factors, such as variation in local transition supports or differences in district practices, may also influence exit timing. Future research should examine factors such as stigma, resource inadequacies, and transition planning to better understand these dynamics.

As autism’s definition broadens to include a wider spectrum of abilities, the diversity in educational needs has grown (Happé & Frith, 2020). It is also important to note that the IDEA autism category may include students both with and without co-occurring intellectual disability, which can reflect different support needs and educational trajectories that cannot be fully distinguished within the aggregated administrative data used in this study. Some autistic students graduate on time with sufficient support, while others require extended educational services. Over half of autistic young adults face severe challenges during their transition to postsecondary life, often more so than their non-disabled peers (Roux et al., 2020; Ruble et al., 2019). Many do not continue with further education or employment and lack sufficient transition plans, as documented in prior research. Within this broader context, strengthening transition frameworks may help address the “services cliff,” although this study does not directly examine these mechanisms.

The variability in exit ages and outcomes underscores the need for adult service systems, such as state Medicaid programs and Vocational Rehabilitation services (VR), to support students transitioning out of high school effectively. Differences across states may also reflect variation in state education policies, graduation requirements, or the maximum age of IDEA eligibility, although these policy mechanisms cannot be directly examined using the available administrative data. Weak links between postsecondary programs and transitioning adolescents exacerbate these challenges. Some state initiatives aim to engage graduates and ensure service continuity, but they are often poorly documented and underfunded (Office of Developmental Programs, 2022). Enhancing transition planning and inter-system coordination may support continuity between education systems and adult service systems following school exit (Shea et al., 2022).

This study has several limitations that provide important context for interpreting the findings and identifying areas for future research. Variability in the implementation of federal special education requirements across states posed a significant limitation, particularly concerning certificate programs. The data did not include detailed information about how certificates are awarded, the criteria for earning a certificate versus a diploma, or how these criteria differ by state and district. This gap limits our ability to understand the potential differences in outcomes for students exiting with certificates compared to diplomas, especially for autistic students and those with intellectual disabilities who are more likely to receive certificates. Additionally, inconsistencies in state data availability, sample sizes, and reporting quality may have influenced the results, potentially underrepresenting certain populations or exit patterns. Variability in state reporting practices may also affect comparability across states and years, as differences in classification, reporting thresholds, or data suppression could influence observed patterns independent of true differences in student outcomes. For example, incomplete or missing data in some states could skew findings toward states with more comprehensive reporting. Because the analysis relies on aggregated administrative data, the results should also be interpreted cautiously to avoid ecological fallacy, in which state-level patterns may not reflect individual experiences or decisions. Future research linking IDEA administrative data with state policy characteristics or other contextual indicators may help clarify how policy environments influence high school exit patterns. Addressing these gaps through standardized data collection would improve the accuracy and comparability of future analyses.

This study also excluded autistic individuals who are not identified as receiving special education services, such as those in mainstream education or not enrolled, which may have led to missing data and underrepresentation of diverse experiences. Furthermore, while the findings reflect patterns among students exiting special education under IDEA, they may not generalize to students in private schools, non-IDEA programs, or other countries. Future research should examine these contexts and incorporate demographic variables such as race, ethnicity, and parental education to uncover disparities and better understand diverse challenges. Investigating the interplay between race and disability, in particular, could reveal systemic inequities in access to extended educational services. By addressing these limitations, future studies can enhance understanding of high school exit patterns and inform targeted strategies for improving transition planning and post-school outcomes for autistic students and others with disabilities.

## 5. Conclusions

As the definition of autism broadens to include a wider range of cognitive abilities and challenges, educational and transition services must evolve to meet these diverse needs effectively. This study highlights the different paths autistic students take—some graduate with peers, while others need extended support up to the IDEA’s age limits. These varied outcomes highlight the importance of coordination between education systems and adult service programs to support students transitioning out of special education. Ensuring adequate resources for transition services remains an important policy consideration, as prior research suggests that funding levels can influence the capacity of schools and service systems to support students as they move beyond high school (Kolbe et al., 2022). Together, these findings underscore the importance of aligning education and adult service systems to support autistic students during the transition to adulthood.

## Figures and Tables

**Table 1:** Average age (in years) when exiting special education for those graduating with a diploma in 2019, by state.

State (Max Age Eligible)	All Disabilities	Autism	Intellectual Disability
United States	17.7	17.9	18.6
Alabama (21)	17.7	17.5	18.0
Alaska (22)	17.7	17.9	18.5
Arizona (21)	17.6	17.7	18.3
Arkansas (21)	17.7	17.6	17.8
California (21)	17.5	17.4	17.7
Colorado (21)	17.9	18.4	19.1
Connecticut (21)	17.5	18.3	19.4
Delaware (20)	17.8	17.8	18.2
District of Columbia	17.4	17.4	17.4
Florida (21)	18.5	18.6	19.0
Georgia (21)	17.7	18.0	18.9
Hawaii (20)	17.2	17.3	17.4
Idaho (21)	17.5	17.7	17.9
Illinois (21)	17.7	18.2	18.8
Indiana (22)	17.8	17.7	18.1
Iowa (21)	17.7	NA	NA
Kansas (21)	17.7	18.1	18.6
Kentucky (21)	17.7	17.6	17.9
Louisiana (21)	NA	NA	NA
Maine (19)	NA	NA	NA
Maryland (21)	17.4	17.5	17.8
Massachusetts (21)	17.4	17.4	17.8
Michigan (25)	17.4	17.4	17.6
Minnesota (21)	17.9	18.3	19.1
Mississippi (20)	NA	NA	NA
Missouri (20)	17.7	17.8	18.0
Montana (18)	17.4	17.3	17.8
Nebraska (20)	18.2	18.6	19.3
Nevada (21)	17.3	17.2	17.4
New Hampshire (21)	NA	NA	NA
New Jersey (21)	18.5	19.4	19.4
New Mexico (21)	17.6	17.5	18.0
New York (20)	17.3	17.3	18.1
North Carolina (20)	17.8	17.7	18.1
North Dakota (21)	18.4	18.8	19.7
Ohio (21)	17.6	17.8	18.0
Oklahoma (21)	NA	NA	NA
Oregon (20)	17.6	18.0	18.5
Pennsylvania (21)	17.8	18.2	18.9
Rhode Island (21)	18.4	18.6	19.8
South Carolina (21)	17.6	17.5	18.0
South Dakota (21)	17.9	18.3	18.7
Tennessee (21)	17.6	17.6	17.9
Texas (21)	17.7	17.7	18.6
Utah (22)	17.5	17.8	18.5
Vermont (21)	NA	NA	NA
Virginia (21)	17.5	17.5	18.1
Washington (21)	18.8	19.3	20.2
West Virginia (21)	17.7	17.7	17.9
Wisconsin (21)	17.5	17.9	18.6
Wyoming (21)	18.8	19.0	NA

Note. States with “NA” average ages either did not have data available or count data was not provided because of small cell sizes. The three highlighted U.S. states had an average age of graduating with a diploma of 19 years or older.

## Data Availability

Data used in this study are publicly available data through the US Department of Education (https://data.ed.gov/dataset, accessed on 1 September 2025).

## Abbreviations

The following abbreviations are used in this manuscript:

IDEA	Individuals with Disabilities Education Act
IEP	Individualized Education Program
